# High folic acid consumption leads to pseudo-MTHFR deficiency, altered lipid metabolism, and liver injury in mice^1^,^2^,^3^,^4^,^5^

**DOI:** 10.3945/ajcn.114.086603

**Published:** 2015-01-07

**Authors:** Karen E Christensen, Leonie G Mikael, Kit-Yi Leung, Nancy Lévesque, Liyuan Deng, Qing Wu, Olga V Malysheva, Ana Best, Marie A Caudill, Nicholas DE Greene, Rima Rozen

**Affiliations:** 1 From the Departments of Human Genetics and Pediatrics, McGill University, and the Montreal Children’s Hospital site of the McGill University Health Centre Research Institute, Montreal, Quebec, Canada (KEC, LGM, NL, LD, QW, and RR); Developmental Biology and Cancer Programme, Institute of Child Health, University College London, London, United Kingdom (K-YL and NDEG); the Division of Nutritional Sciences and Genomics, Cornell University, Ithaca, NY (OVM and MAC); and the Department of Mathematics and Statistics, McGill University, Montreal, Quebec, Canada (AB).

**Keywords:** choline metabolism, folic acid, lipid metabolism, liver, methylenetetrahydrofolate reductase

## Abstract

**Background:** Increased consumption of folic acid is prevalent, leading to concerns about negative consequences. The effects of folic acid on the liver, the primary organ for folate metabolism, are largely unknown. Methylenetetrahydrofolate reductase (MTHFR) provides methyl donors for S-adenosylmethionine (SAM) synthesis and methylation reactions.

**Objective:** Our goal was to investigate the impact of high folic acid intake on liver disease and methyl metabolism.

**Design:** Folic acid–supplemented diet (FASD, 10-fold higher than recommended) and control diet were fed to male *Mthfr*^+/+^ and *Mthfr*^+/−^ mice for 6 mo to assess gene-nutrient interactions. Liver pathology, folate and choline metabolites, and gene expression in folate and lipid pathways were examined.

**Results:** Liver and spleen weights were higher and hematologic profiles were altered in FASD-fed mice. Liver histology revealed unusually large, degenerating cells in FASD *Mthfr*^+/−^ mice, consistent with nonalcoholic fatty liver disease. High folic acid inhibited MTHFR activity in vitro, and MTHFR protein was reduced in FASD-fed mice. 5-Methyltetrahydrofolate, SAM, and SAM/S-adenosylhomocysteine ratios were lower in FASD and *Mthfr*^+/−^ livers. Choline metabolites, including phosphatidylcholine, were reduced due to genotype and/or diet in an attempt to restore methylation capacity through choline/betaine-dependent SAM synthesis. Expression changes in genes of one-carbon and lipid metabolism were particularly significant in FASD *Mthfr*^+/−^ mice. The latter changes, which included higher nuclear sterol regulatory element-binding protein 1, higher *Srepb2* messenger RNA (mRNA), lower farnesoid X receptor (*Nr1h4*) mRNA, and lower *Cyp7a1* mRNA, would lead to greater lipogenesis and reduced cholesterol catabolism into bile.

**Conclusions:** We suggest that high folic acid consumption reduces MTHFR protein and activity levels, creating a pseudo-MTHFR deficiency. This deficiency results in hepatocyte degeneration, suggesting a 2-hit mechanism whereby mutant hepatocytes cannot accommodate the lipid disturbances and altered membrane integrity arising from changes in phospholipid/lipid metabolism. These preliminary findings may have clinical implications for individuals consuming high-dose folic acid supplements, particularly those who are MTHFR deficient.

## INTRODUCTION

Folates are required for nucleotide synthesis and S-adenosylmethionine (SAM)^6^–dependent methylation reactions (**Supplemental Figure 1**). Folic acid is a synthetic folate added to fortified foods and nutritional supplements. Fortification has reduced the incidence of neural tube defects (1, 2) and other disorders (3), but there are concerns that high folic acid intake, due to fortification and vitamin supplementation, may negatively affect health (4, 5). Recent National Health and Nutrition Examination Survey data showed that ∼25% of children and 5% of adults older than 50 y consume more than the recommended upper limit for folic acid (6–8). Unmetabolized folic acid (UFA) appears in plasma after consuming the recommended daily intake of 400 μg/d (9). However, most supplements contain ≥400 μg, and supplement users in the Framingham cohort were 2.3-fold more likely to have high circulating UFA than nonusers (10). Supplements containing 4000–5000 μg/d are recommended to women with high-risk pregnancies (11, 12). In an elderly population administered 5000 μg/d for 3 wk, UFA was found in 100% of the study group (compared with 26% at baseline), and concentrations rose from 0.8% to 15% of total folates (13).

The possible negative effects of high folic acid consumption have prompted investigations into its impact on health. We found that pregnancy losses and embryonic heart defects were more common in mice consuming a diet high in folic acid (14). Other researchers have suggested links between circulating UFA and lower cognitive test scores (15), as well as impaired immune function (16) in humans.

The liver is an important organ in folate metabolism; most folate-dependent enzymes and the majority of folate are found there (17). Nonetheless, the effect of high folic acid consumption on the liver has not been well examined. UFA may affect the liver through mechanisms such as enzyme inhibition, either directly (18–23) or through its metabolites (24–26). The impact of UFA may be modulated by genetic variants in these enzymes.

Methylenetetrahydrofolate reductase (MTHFR) produces methyltetrahydrofolate for remethylation of homocysteine to methionine by methionine synthase (Supplemental Figure 1). In the liver and kidney, an alternate pathway uses betaine to remethylate homocysteine through betaine-homocysteine methyltransferase (27) linking choline metabolism to folate metabolism. We observed increased flux through betaine-homocysteine methyltransferase when folate metabolism was disturbed in humans (28, 29) and mice (28, 30) due to MTHFR mutation (28) or low dietary folate (30). We found that male mice relied more on the betaine pathway than did female mice, resulting in greater susceptibility to hepatic steatosis (30).

In this report, we examine the effects of high folic acid consumption on the liver. To investigate diet and gene variant interactions, we used mice with mild MTHFR deficiency, a model for the *MTHFR* 677 C>T polymorphism, the most studied variant in folate metabolism with 5–20% homozygosity (31).

## METHODS

### Mice, diets, and sample collection

All experiments were performed within the guidelines of the Canadian Council on Animal Care and approved by the Animal Care Committee of the Research Institute of the McGill University Health Centre. BALB/c *Mthfr*^+/+^ and *Mthfr*^+/−^ males (32) were placed on control (CD) and 10× folic acid–supplemented (FASD) amino acid–defined diets (Harlan) at weaning. CD (TD.01369) contained 2 mg/kg folic acid, the Recommended Dietary Allowance (RDA) for rodents set by the American Institute of Nutrition (33), whereas FASD (TD.09258) contained 20 mg/kg diet (10× RDA); both diets contained 2.5 g/kg choline bitrate, 3.3 g/kg L-methionine, and 1% succinylsulfathiazole (to inhibit folate production by intestinal flora). Litters were randomly assigned to CD or FASD to produce a total of 7–8 mice in each of the 4 diet/genotype experimental groups (29 total). Mice were housed under specific pathogen-free conditions in a controlled environment (12 h/12 h light/dark cycle, 18–24°C) with ad libitum access to food and water.

After 6 mo on diets, mice were sacrificed in random order by CO_2_ asphyxiation and body weights recorded. Blood was collected by cardiac puncture in potassium-EDTA–coated tubes. Complete blood counts were performed at the Animal Resource Center, McGill University. Plasma was obtained by centrifugation at 3000 × *g* for 7 min and stored at −75°C or below. Tissues were collected, weighed, and rinsed with cold phosphate-buffered saline. The entire left lobe of the liver was fixed in 4% paraformaldehyde for 1 d and stored at 4°C in 70% ethanol; the remaining tissue was snap frozen on dry ice and stored at −75°C or below.

### Histologic examination

Fixed liver was embedded in paraffin and cut in 5-μm sections. Sections were stained with hematoxylin and eosin or Masson’s trichrome stain (Polysciences Inc.). Two hematoxylin and eosin–stained sections per mouse were examined in random order at 100×, 200×, and 400× magnification (2 fields per section) and scored for the presence of degenerating cells and lipid droplets by a blinded observer and confirmed by a second observer. Degenerating cells were enlarged, contained strands of eosinophilic material, and had clearing of the cytoplasm. Preliminary inspection showed that the liver sections fell into 2 groups: *1*) no or small localized foci of degenerating cells or *2*) large numbers of degenerating cells affecting at least half of the section. Therefore, livers were scored as containing degenerating cells if more than half of the hepatocytes in the field at 200× were affected in at least 3 of the 4 fields examined (i.e., small localized foci were not considered meaningful). Accumulation of lipid droplets (microvesicular and macrovesicular) was graded as described (30).

### MTHFR inhibition assays

Brain and liver extracts for enzyme assays were prepared from ∼50 mg frozen tissue under native conditions [lysis buffer: 50 mmol/L potassium phosphate, 0.3 mmol/L EDTA, supplemented with protease (Roche) and phosphatase inhibitors (Thermo Scientific)] by using a bead mill and cleared by centrifugation. Protein concentration was determined by Bradford assay by using bovine serum albumin as a standard. Activity assays were performed as in Goyette et al. (34) with the following modifications: the assay mix was modified to contain 200 μmol/L substrate and 30 μmol/L flavin adenine dinucleotide. Then, 1.4 mmol/L folic acid in 3.5 mmol/L NaOH was added to the assay to final concentrations of 0, 250, 500, and 750 μmol/L, and 3.5 mmol/L NaOH was added to each reaction to equalize the final concentration of NaOH in each reaction. Assays were performed in triplicate. Results are expressed as percent MTHFR activity at 0 μmol/L folic acid and reported as the mean ± SEM of 2 (brain) or 3 (liver) separate experiments.

### Western blotting

Total liver extracts were prepared as for MTHFR assays (see previous paragraph). Nuclear and cytoplasmic fractions were isolated by using the NE-PER Nuclear Protein Extraction Kit (Thermo Scientific) following the manufacturer’s instructions. Protein concentration was determined by Bradford assay by using bovine serum albumin as a standard. Western blotting was performed with conventional methods by using 25 μg total or cytoplasmic protein or 15 μg nuclear protein. Primary antibodies were sterol regulatory element-binding protein (SREBP-1; Santa Cruz Biotechnology), cyclic AMP-responsive element-binding protein (Cell Signaling Technology), β-actin (Sigma-Aldrich), and MTHFR (35). Secondary antibody was horseradish peroxidase–coupled anti–rabbit IgG (GE Healthcare). Bands were visualized with Amersham ECL Prime Western Blotting Detection Reagent (GE Healthcare) and film exposure and quantified by densitometry by using Quantity One 1-D Analysis Software version 4.6.9 (Bio-Rad). Results were normalized to the β-actin loading control and reported relative to the mean value for CD-fed *Mthfr*^+/+^ mice, which was standardized to a reference value of 1.

### Alkaline phosphatase treatment

Alkaline phosphatase treatment of protein extracts was adapted from Yamada et al. (36). Briefly, total liver extract was isolated in RIPA lysis buffer (denaturing conditions: 150 mmol/L NaCl, 50 mmol/L Tris-HCl, 1 mmol/L EDTA, 1% NP-40, 0.5% sodium deoxycholate, and 0.1% SDS) supplemented with protease inhibitors. Then, 50 μg protein was treated with 25 units of calf intestinal alkaline phosphatase (Roche), incubated at 37°C for 1 h, and analyzed for MTHFR by Western blotting.

### Folates

The distribution of folates in frozen liver [specifically: folic acid, dihydrofolate, tetrahydrofolate (THF), methenylTHF, methyleneTHF, methylTHF, and formylTHF] was determined by liquid chromatography–electrospray ionization tandem mass spectrometry. Samples were prepared as described in Christensen et al. (37). Analytes were resolved by reversed-phase chromatography and measured as described previously (37–39). Total folates are reported as nmol/g liver; the concentration of individual folate forms was normalized to total folate concentration and reported as the percentage of total folates to allow comparison of distributions.

### Choline metabolites

The concentrations of choline metabolites in frozen liver were measured by liquid chromatography–mass spectrometry. Choline, betaine, glycerophosphocholine, phosphocholine (PCho), phosphatidylcholine (PtdCho), sphingomyelin (SM), and lysophosphatidylcholine (LysoPtdCho) were measured by using the method in Koc et al. (40), with modifications (29, 41). SAM and S-adenosylhomocysteine (SAH) were determined by liquid chromatography–mass spectrometry as described (42), with modifications based on our instrumentation (43).

### Quantitative reverse transcriptase–polymerase chain reaction

Total RNA was extracted from ∼10 mg frozen liver by using the RNeasy Mini kit with on-column DNase I treatment (Qiagen). Complementary DNA synthesis was performed as in Knock et al. (44). Quantitative reverse transcriptase–polymerase chain reaction (RT-PCR) was performed by using Platinum SYBR Green qPCR SuperMix-UDG master mix (Invitrogen) on a Lightcycler LC480 (Roche Diagnostics). The following reference genes were evaluated: β-actin (*Actb*), β-2-microglobulin (*B2m*), succinate dehydrogenase (*Sdha*), and tyrosine 3-monooxygenase/tryptophan 5-monooxygenase activation protein, zeta polypeptide (*Ywhaz*) [all primers as in Christensen et al. (30)] and glyceraldehyde-3-phosphate dehydrogenase (*Gapdh*) [primers as in Leclerc et al. (45)]. The 3 most stably expressed reference genes were determined (*Gapdh*, *Actb*, and *B2m*) and used to calculate one normalization factor for target gene expression by geNorm v.3.4 (Ghent University Hospital Center for Medical Genetics) (46). Primers for the target genes betaine-homocysteine methyltransferase (*Bhmt*) and methionine synthase (*Mtr*) were reported in Christensen et al. (30). Primers for Bcl2-like 1 (*Bcl-XL*) and BCL2-antagonist/killer 1 (*Bak*) were reported in Garcia-Crespo et al. (47). All other target gene primers [choline dehydrogenase (*Chdh*); cytochrome P450, family 7, subfamily a, polypeptide 1 (*Cyp7a1*); fatty acid desaturase 2 (*Fads2*); methionine adenosyltransferase I, α (*Mat1a*); nuclear receptor subfamily 1, group H, member 4 (*Nr1h4*); farnesoid X receptor (FXR); phosphatidylethanolamine N-methyltransferase (*Pemt*); patatin-like phospholipase domain containing 2 (*Pnpla2*); peroxisome proliferator-activated receptor α (*Ppara*); stearoyl–coenzyme A desaturase 1 (*Scd1*); sterol regulatory element-binding transcription factor 1 (*Srebp1*); and sterol regulatory element-binding transcription factor 2 (*Srebp2*)] were designed by using Primer-BLAST (National Center for Biotechnology Information) (48). For a complete list of primer sequences and reaction conditions, see **Supplemental Table 1**.

### Quantitative CpG methylation analysis

Using a panoply of analysis tools, CpG islands or similar features could not be identified in the *Cyp7a1* gene or its vicinity (data not shown). However, 2 CpG sites in the *Cyp7a1* gene were identified as potentially differentially methylated because of a DNA region reported to contain methylation flags (49) and were assessed by bisulfite pyrosequencing as previously described (45). Briefly, extracted DNA was subjected to bisulfite treatment by using the Epitect Bisulfite Kit (Qiagen). Primers for pyrosequencing of *Cyp71a* were designed with PyroMark Assay Design 2.0 software (Qiagen) (5′-biotinylated oligonucleotide: 5′-ACCTTCTCCATATCATCAAAAATAAAAAAT-3′, sense PCR primer: 5′-AAGTTAGGGAAAGGTTGGTTGAGAG-3′, sequencing primer: 5′-GAATTTGTATATGAGGGATTAG-3′). Pyrosequencing was performed on the PyroMark Q24 Platform (Qiagen). Data were interpreted with PyroMark Q24 2.0.6 analysis software.

### Caspase 3/7 activity

Total liver extracts were prepared as for MTHFR assays (see above). Caspase activity was measured with the Caspase-Glo 3/7 Assay System (Promega) by using 2 μg total liver extract in 50 μL assay reagent following the manufacturer’s instructions. Luminescence was measured with a Glo-max Multi Detection Microplate reader (Promega).

### Statistical methods

All results are expressed as mean ± SE. Because this preliminary study involved the use of a new diet, with unpredictable effects, we did not perform a sample size calculation. Sample sizes were determined based on previous experience with these methods in our mouse models (30, 37). Unless noted, data were analyzed by using 2-factor ANOVA with diet and genotype as the independent variables, followed by post hoc analysis by Tukey to correct for multiple comparisons if the interaction term was significant or borderline significant (*P* ≤ 0.058 where indicated). Effect of folic acid on MTHFR activity was assessed by using 1-factor ANOVA for comparison with the 0-μmol/L folic acid control, with post hoc analysis by Tukey. Incidence of degenerating hepatocytes was assessed by exact binary logistic regression for diet and genotype with individual mice as the unit of analysis by using SAS version 9.4 (SAS Institute). Statistical outliers were identified by using Grubb’s test (QuickCalcs; GraphPad Software) and removed from the analysis. No other corrections were made for multiple testing. Results of statistical tests were considered significant at *P* ≤ 0.05 and borderline significant up to *P* ≤ 0.075 where indicated. Statistical analyses were carried out by using SPSS version 20.0.0 (SPSS Inc.) unless noted.

## RESULTS

### FASD consumption affects organ weights and hematology

Liver and spleen weights (adjusted for body weight) of mice fed FASD were significantly higher than those of mice fed CD (**Table 1**). These particular organs may be sensitive to folate intake due to their roles in folate metabolism and hematopoiesis. White blood cell counts (specifically lymphocytes) were significantly higher in *Mthfr*^+/−^ mice compared with *Mthfr*^+/+^, but this difference was not affected by diet (**Table 2**). In contrast, red blood cell (RBC) counts and related hematologic parameters were significantly affected by both diet and *Mthfr* genotype, with more marked changes in the FASD-fed *Mthfr*^+/−^ group (Table 2). The reduction in RBC counts, hematocrit, and hemoglobin because of FASD and genotype and the larger mean corpuscular volume because of FASD are reminiscent of the megaloblastic anemia associated with folate deficiency.

**TABLE 1:** Mice fed the FASD have higher liver and spleen weights^1^

	CD		FASD		2-Factor ANOVA *P* value		
Characteristic	*Mthfr*^+/+^	*Mthfr*^+/−^	*Mthfr*^+/+^	*Mthfr*^+/−^	Genotype	Diet	Interaction
Body weight, g	29.8 ± 1.2^2^	28.2 ± 1.2	28.6 ± 0.9	29.7 ± 1.2	0.831	0.897	0.224
Organ weight (as % body weight)							
Liver	4.02 ± 0.20	4.00 ± 0.23	4.37 ± 0.16	4.95 ± 0.07	0.118	0.001	0.105
Spleen	0.28 ± 0.01	0.31 ± 0.02	0.34 ± 0.03	0.36 ± 0.04	0.389	0.046	0.970
Brain	1.54 ± 0.07	1.58 ± 0.04	1.58 ± 0.04	1.47 ± 0.02	0.451	0.469	0.128

1 *n* = 6–8 per group. CD, control diet; FASD, folic acid–supplemented diet.

2 Mean ± SE (analyzed by 2-factor ANOVA; all such values).

**TABLE 2:** Complete blood counts of CD- and FASD-fed *Mthfr*^+/+^ and *Mthfr*^+/−^ mice reveal hematologic changes because of both diet and MTHFR deficiency^1^

	CD		FASD		2-Factor ANOVA *P* value		
Characteristic	*Mthfr*^+/+^	*Mthfr*^+/−^	*Mthfr*^+/+^	*Mthfr*^+/−^	Genotype	Diet	Interaction
Red blood cells							
Count, ×10^12^/L	12.82 ± 0.28^2^	11.80 ± 0.50	11.55 ± 0.84	9.67 ± 0.75	0.036	0.015	0.510
Hematocrit, L/L	0.624 ± 0.015	0.573 ± 0.023	0.571 ± 0.040	0.488 ± 0.035	0.040	0.036	0.611
Hemoglobin, g/L	196 ± 3	189 ± 8	191 ± 5	163 ± 10	0.020	0.038	0.165
MCV, fL	48.6 ± 0.3	48.7 ± 0.2	49.4 ± 0.4	50.6 ± 0.5	0.082	0.001	0.167
MCH, pg	15.3 ± 0.2	16.0 ± 0.1	15.9 ± 0.3	17.0 ± 0.3	0.003	0.012	0.410
MCHC, g/L	315 ± 6	329 ± 3	320 ± 7	334 ± 3	0.010	0.332	0.989
White blood cells							
Count, ×10^9^/L	5.9 ± 0.8	7.8 ± 0.9	6.3 ± 0.9	8.5 ± 0.8	0.026	0.501	0.862
Neutrophils, %	32 ± 4	28 ± 5	32 ± 2	26 ± 1	0.167	0.696	0.774
Lymphocytes, %	68 ± 4	72 ± 5	68 ± 2	74 ± 1	0.183	0.764	0.816
Neutrophils, ×10^9^/L	1.45 ± 0.13	2.01 ± 0.21	1.98 ± 0.29	2.35 ± 0.29	0.081	0.098	0.719
Lymphocytes, ×10^9^/L	3.95 ± 0.47	5.78 ± 0.97	4.34 ± 0.67	6.15 ± 0.58	0.015	0.595	0.992
Platelets, ×10^9^/L	865 ± 70	899 ± 51	901 ± 41	1032 ± 31	0.122	0.114	0.352

1 *n* = 6–8 per group. CD, control diet; FASD, folic acid–supplemented diet; MCH, mean corpuscular hemoglobin; MCHC, mean corpuscular hemoglobin concentration; MCV, mean corpuscular volume.

2 Mean ± SE (analyzed by 2-factor ANOVA; all such values).

### Liver histologic examination reveals that the combination of MTHFR deficiency and high folic acid consumption causes liver injury

**FIGURE 1:**
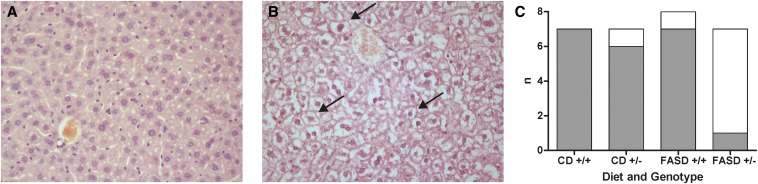
High folic acid consumption in *Mthfr*^+/−^ mice may cause degeneration of hepatocytes. Hematoxylin and eosin–stained liver sections from (A) CD^+/−^ and (B) FASD^+/−^ mice (400× magnification). Most cells in FASD *Mthfr*^+/−^ liver appear unusually large with clear patches in the cytoplasm consistent with hepatocyte degeneration. In contrast, most livers of the other 3 groups appeared normal or had only isolated abnormal cells. Examples of these unusual cells are indicated by arrows. (C) Incidence of widespread degenerating hepatocytes in diet/genotype groups (*n* = 7–8 per group). Both diet (*P* = 0.016; estimated OR: 25.0) and genotype (*P* = 0.009; estimated OR: 28.9) had significant effects on the incidence of widespread degeneration (exact binary logistic regression). CD, control diet; FASD, folic acid–supplemented diet.

Examination of hematoxylin and eosin–stained sections revealed histologic changes associated with nonalcoholic fatty liver disease (NAFLD). Considerable numbers of unusually large cells with vacuolation of the cytoplasm, consistent with hydropic degeneration or hepatocyte ballooning, were observed (**Figure 1**). Both diet and genotype significantly affected the incidence of degenerating cells. However, it is clear from inspection of the data (Figure 1C) that these results are driven by the FASD *Mthfr^+/−^* group, as 6 of 7 of FASD *Mthfr^+/−^* livers presented with this phenotype, compared with a maximum of 1 of 7 in the other groups. Hydropic degeneration, caused by accumulation of fluid in the cytoplasm, is associated with disturbed membrane integrity (50). Changes in membrane integrity can result from disruption of phospholipid metabolism, because phospholipids, especially phosphatidylcholine (PtdCho), are critical components of membranes (51).

Lipid droplets were also observed, as previously reported in *Mthfr*^+/−^ mice and in mice fed control or low-folate diets (30, 52). The incidence and degree of steatosis in CD-fed mice was consistent with our previous observations of mice fed CD for 12 mo (30). The accumulation of lipids did not appear to be different between the CD^+/+^, CD^+/−^, and FASD^+/+^ groups (data not shown). Accurate evaluation of lipid droplet accumulation was extremely difficult for livers with large numbers of degenerated cells; therefore, the extent of steatosis in FASD^+/−^ mice could not be determined. It is possible that lipid accumulation is a precursor to degeneration; additional experimentation would be required to assess. Masson’s trichrome–stained sections were also examined; no unusual collagen staining was observed (data not shown), indicating that liver damage in these mice had not yet progressed to fibrosis.

### MTHFR activity is inhibited by folic acid in vitro, and immunoreactive protein levels are reduced in FASD-fed mice

**FIGURE 2:**
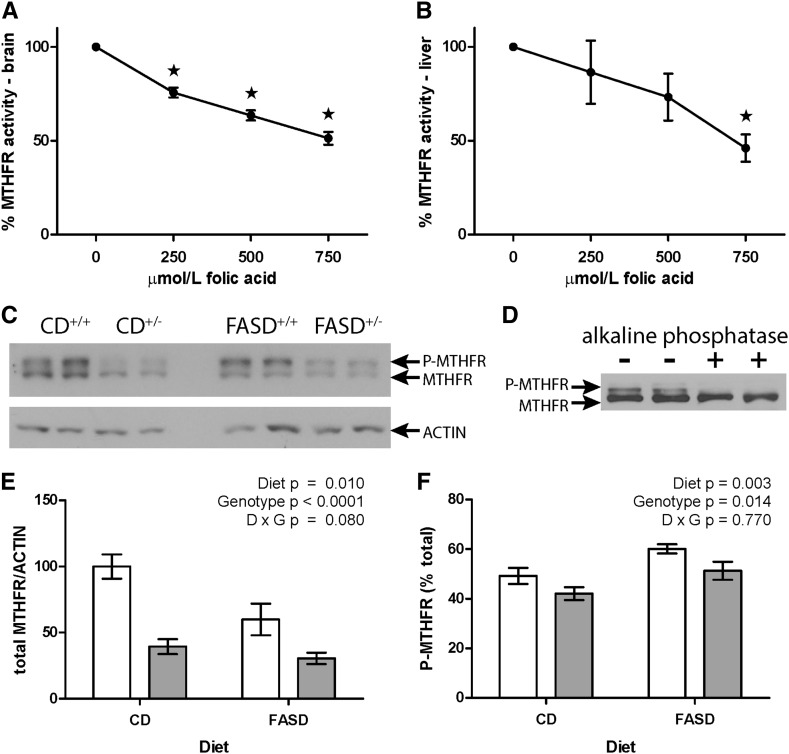
MTHFR activity is inhibited by folic acid in vitro, and immunoreactive protein levels are reduced in FASD-fed mice. MTHFR enzyme activity assayed in crude brain (A) and liver (B) extracts is reduced with increasing amounts of folic acid. Assays were performed in triplicate and reported as the means ± SEMs of 2 (brain) or 3 (liver) separate experiments. *Significantly different from 0 μmol/L folic acid, 1-factor ANOVA, Tukey post hoc, *P* < 0.05. (C) Representative Western blot of liver extracts from CD-fed and FASD-fed *Mthfr*^+/+^ and *Mthfr*^+/−^ mice. MTHFR appears as a doublet of the phosphorylated and nonphosphorylated 70-kDa isoform. (D) Alkaline phosphatase treatment of liver extracts confirms that the upper band is the phosphorylated 70-kDa isoform of MTHFR (experiment performed in duplicate). (E) Quantification of MTHFR immunoreactive protein shows that MTHFR expression is reduced ∼40% in *Mthfr*^+/+^ mice fed FASD compared with CD; MTHFR expression is reduced ∼60% in *Mthfr*^+/−^ mice compared with *Mthfr*^+/+^ mice (*n* = 5 per group, mean ± SEM, 2-factor ANOVA). (F) The proportion of phosphorylated MTHFR is significantly lower because of the *Mthfr*^+/−^ genotype but higher because of the FASD (*n* = 5 per group, mean ± SEM, 2-factor ANOVA). White bars: *Mthfr*^+/+^; gray bars: *Mthfr*^+/−^. CD, control diet; D, diet; FASD, folic acid–supplemented diet; G, genotype; MTHFR, methylenetetrahydrofolate reductase.

Folic acid has previously been reported to inhibit MTHFR activity in crude brain extract (21). Because our extraction and assay methods differed from that report, we assessed the inhibition of MTHFR by folic acid in crude brain extract (**Figure 2A**). MTHFR was also inhibited in crude liver extract (Figure 2B). Half-maximal inhibition of MTHFR was observed at 750 μmol/L folic acid (<4-fold substrate concentration), suggesting that UFA could contribute to MTHFR deficiency.

Total immunoreactive MTHFR protein was reduced in *Mthfr*^+/−^ livers as expected. However, MTHFR protein was also affected by FASD (Figure 2C,E). The upper band in Figure 2C was identified as the phosphorylated 70-kDa isoform of MTHFR (36) by treatment with alkaline phosphatase (Figure 2D). The relative proportion of phosphorylated and nonphosphorylated MTHFR was sensitive to both diet and MTHFR deficiency (Figure 2C,F). The percentage of phosphorylated MTHFR, which is less active than the nonphosphorylated form (36), rose significantly in FASD^+/+^ compared with CD^+/+^. Overall, the immunoblotting data suggest that MTHFR activity was reduced in the liver of FASD-fed mice through reduced protein levels and greater phosphorylation.

### Methylation capacity is reduced in the livers of FASD-fed mice

**FIGURE 3:**
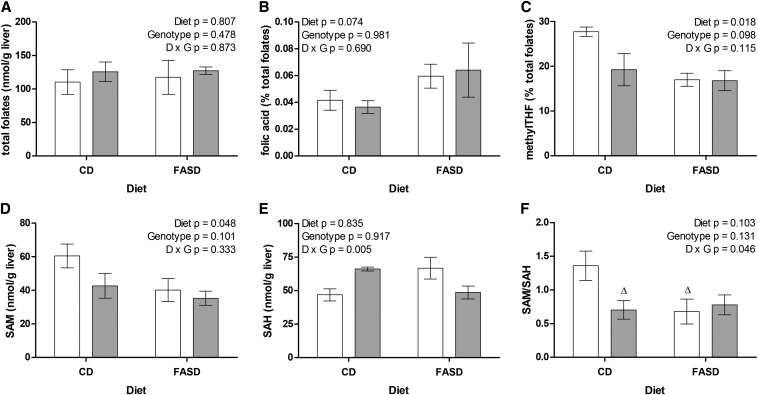
High folic acid consumption reduces methylTHF and methylation capacity. (A) Total liver folate content did not differ between groups. (B) The proportion of unmetabolized folic acid (percentage of total folates) in liver was ∼60% higher in FASD-fed mice (borderline significant, *P* = 0.074). (C) The proportion of methylTHF was significantly lower in FASD-fed mice. (D) SAM concentrations were significantly lower in FASD-fed mice. (E) There was a significant interaction between the effects of diet and *Mthfr* genotype on SAH concentrations, but there was no significant difference between groups by Tukey post hoc comparisons. (F) Methylation capacity as measured by SAM/SAH ratio. There was a significant interaction between diet and genotype; CD^+/−^ and FASD^+/+^ groups were borderline significantly different from CD^+/+^ by Tukey post hoc analysis (^Δ^*P* = 0.062–0.074). White bars: *Mthfr*^+/+^; gray bars: *Mthfr*^+/−^. *n* = 4–5 per group, mean ± SEM, analyzed by 2-factor ANOVA. CD, control diet; D, diet; FASD, folic acid–supplemented diet; G, genotype; SAH, S-adenosylhomocysteine; SAM, S-adenosylmethionine.

Key folate derivatives were evaluated in the liver (**Figure 3** and **Supplemental Table 2**). There was no significant change in the concentration of total folate due to diet or genotype (Figure 3A), as previously observed in mice fed folate-supplemented diets (53). Therefore, to compare folate distributions, individual concentrations were normalized to the total and expressed as percent total folates. Folic acid in the liver was ∼60% greater in mice fed FASD (Figure 3B). MethylTHF was lower in all groups compared with CD^+/+^ (Figure 3C), consistent with reduced expression and activity of MTHFR (Figure 2). There were no significant changes due to diet or genotype in the proportion of other folates (dihydrofolate, THF, methenylTHF, methyleneTHF, and formylTHF) (Supplemental Table 2). These findings suggest that the substrate of MTHFR, methyleneTHF, is being redistributed among the other nonmethyl forms. High folic acid intake appears to be altering methylation potential, without dramatically changing the availability of folate substrates for nucleotide synthesis and other functions. However, some variability between mice in the levels of non-methylTHF may have contributed to this lack of significance.

The concentration of SAM is affected in the same manner as methylTHF (Figure 3D), demonstrating that reduced MTHFR activity affects the supply of methionine for SAM synthesis. The concentration of SAH did not change due to either diet or genotype alone, although the interaction between those variables was significant (Figure 3E), suggesting that an effect of FASD on SAH levels is genotype dependent. It was not possible to resolve this interaction due to lack of statistical power. However, the more critical indicator of methylation capacity, the SAM/SAH ratio (Figure 3F), was lower in all groups relative to CD^+/+^. The graphs in Figures 2E, 3C, 3D, and 3F have very similar patterns, consistent with reduced MTHFR protein leading to impaired methylTHF production with consequent reduction of SAM and SAM/SAH ratios.

### Expression of methylation cycle genes is altered in FASD-fed mice

**FIGURE 4:**
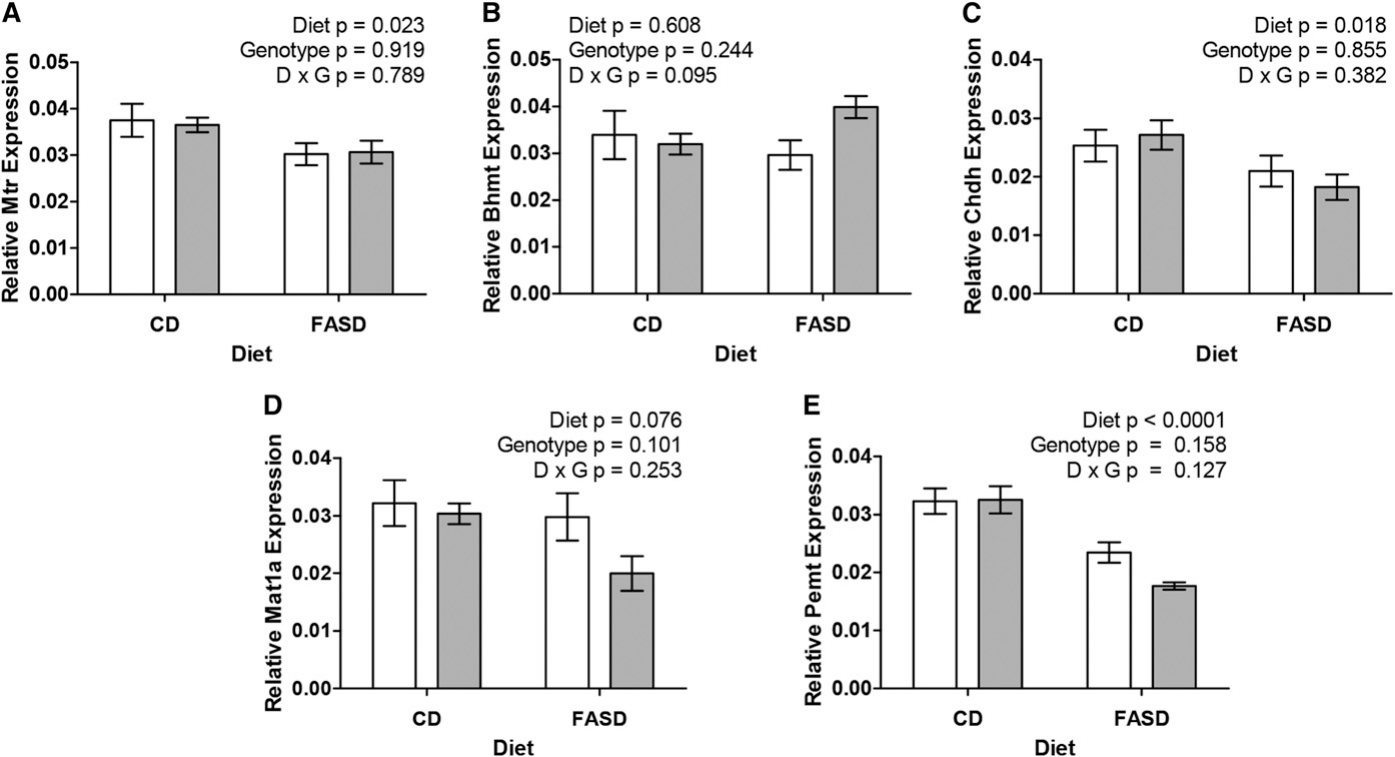
High folic acid consumption reduces messenger RNA levels of genes involved in the methylation cycle. (A) Expression of *Mtr* is reduced in the livers of FASD-fed mice compared with CD-fed mice. (B) Expression of *Bhmt* is unaffected by diet or genotype. (C) *Chdh* expression is reduced because of diet. (D) Expression of *Mat1a* was unchanged by genotype but tended to be lower in mice fed the FASD (*P* = 0.076). (E) Expression of *Pemt,* a major consumer of S-adenosylmethionine, was lower because of the FASD. White bars: *Mthfr*^+/+^; gray bars: *Mthfr*^+/−^. *n* = 4–5 per group, mean ± SEM, analyzed by 2-factor ANOVA. CD, control diet; D, diet; FASD, folic acid–supplemented diet; G, genotype.

The expression of some critical genes in methylation—*Mtr*, *Bhmt*, *Chdh*, *Mat1a*, and *Pemt* (pathways in Supplemental Figure 1)—was evaluated by quantitative RT-PCR. These genes code for enzymes required for the supply and use of SAM in liver. *Mtr* and *Bhmt* catalyze the methylTHF-dependent and betaine-dependent remethylation of homocysteine to methionine, respectively. CHDH catalyzes the first, committed step in the metabolism of choline to betaine. MAT1A synthesizes SAM from methionine and ATP, whereas PEMT produces PtdCho from phosphatidylethanolamine (PE), consuming 3 molecules of SAM. The expression of *Mtr*, *Chdh*, and *Pemt* was significantly reduced in FASD-fed mice; *Mat1a* expression tended to be lower (*P* = 0.076), and *Bhmt* expression did not change (**Figure 4**). Reduced *Mtr* was previously reported in the livers of mice fed a folate-deficient diet (30) and suggests a shift toward folate-independent homocysteine remethylation due to reduced methylTHF substrate. A similar feedback mechanism could be lowering the expression of *Pemt* as a result of reduced SAM.

### Choline and phospholipid metabolite concentrations are changed in *Mthfr*-deficient mice, particularly when fed FASD

**FIGURE 5:**
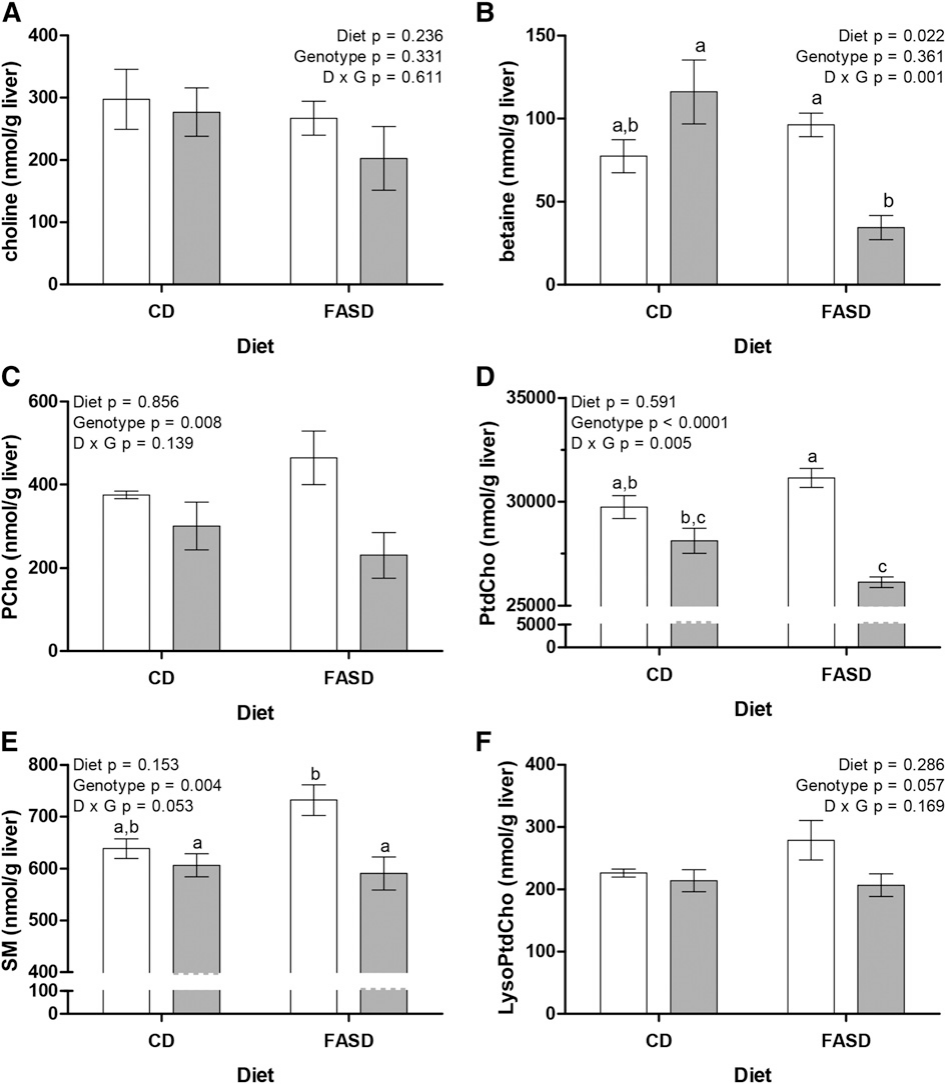
MTHFR deficiency alters choline and phospholipid metabolite concentrations, particularly in FASD-fed mice. The concentration of choline (A) did not change significantly. Betaine concentration (B) was significantly reduced in mice fed the FASD, and there was a significant interaction between the effects of diet and *Mthfr* genotype. (C) PCho was significantly lower in *Mthfr*^+/−^ mice than in *Mthfr*^+/+^ mice. (D) PtdCho concentrations were significantly altered by *Mthfr* genotype, with a significant interaction between diet and genotype. The concentrations of SM (E) and LysoPtdCho (F), products of PtdCho catabolism, were affected by *Mthfr* genotype. (E) SM concentrations were significantly lower in *Mthfr*^+/−^ mice, and there was a borderline significant interaction between diet and genotype (*P* = 0.053). (F) LysoPtdCho tended to be lower in *Mthfr*^+/−^ mice (*P* = 0.057). White bars: *Mthfr*^+/+^; gray bars: *Mthfr*^+/−^. *n* = 4–5 per group, mean ± SEM, analyzed by 2-factor ANOVA. Bars without a common lowercase letter are significantly different by Tukey post hoc, *P* < 0.05. CD, control diet; D, diet; FASD, folic acid–supplemented diet; G, genotype; LysoPtdCho, lysophosphatidylcholine; MTHFR, methylenetetrahydrofolate reductase; PCho, phosphocholine; PtdCho, phosphatidylcholine; SM, sphingomyelin.

Hepatic concentrations of choline and related metabolites were measured (**Figure 5** and Supplemental Table 2). The concentration of choline did not change significantly, consistent with previous observations that choline stores in liver resist depletion (30, 54, 55). Betaine concentrations, on the other hand, dropped significantly as a result of FASD in *Mthfr*^+/−^ mice, suggesting greater use of betaine for homocysteine remethylation when methylTHF abundance is reduced. Concentrations of PCho, a choline storage form and intermediate in the cytidine-diphosphocholine (CDP) choline pathway for PtdCho synthesis, were also significantly lower due to the *Mthfr*^+/−^ genotype, as were the concentrations of PtdCho and its catabolite SM, with borderline significance for another catabolite, LysoPtdCho (*P* = 0.057). These observations suggest that there is greater reliance on choline metabolism to support the methylation cycle when MTHFR is deficient.

### Expression of genes in lipid and cholesterol metabolism is altered by high folic acid consumption, particularly in *Mthfr*-deficient mice

**FIGURE 6:**
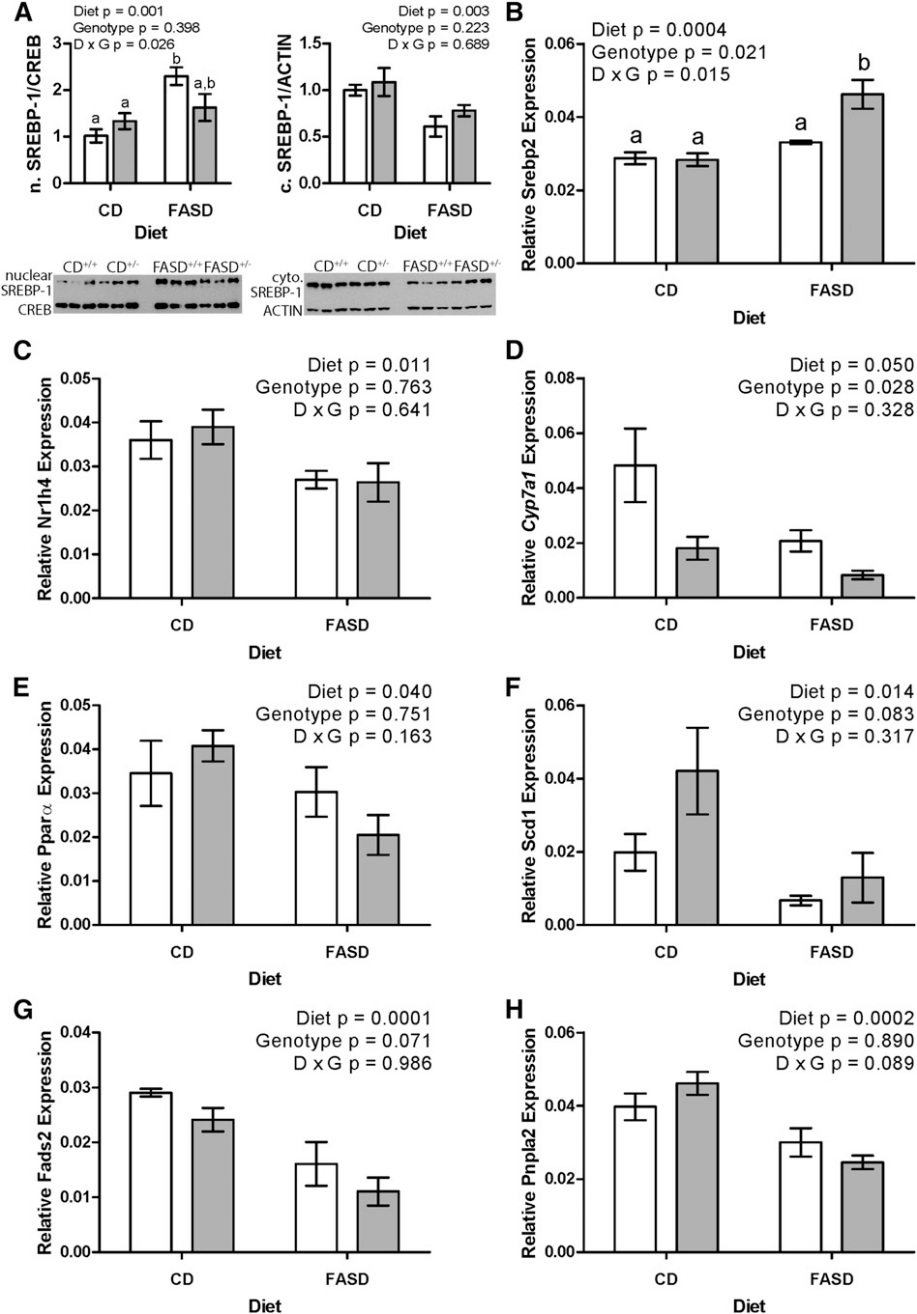
High folic acid consumption alters expression of genes in lipid and cholesterol metabolism, particularly in *Mthfr*^+/−^ mice. (A) Expression of mature SREBP-1 p68 was higher in the nucleus (left) and lower in the cytoplasm (right) of the livers of mice fed the FASD, *n* = 6 per group, with a significant interaction between diet and genotype for nuclear SREBP-1 (left). Representative Western blots are shown below the graphs. (B) *Srebp2* expression was higher because of diet and *Mthfr* genotype, with a significant interaction between diet and genotype. Bars without a common lowercase letter are significantly different by Tukey post hoc, *P* < 0.05. (C) Expression of the transcriptional regulator *Nr1h4* (farnesoid X receptor) was lower in FASD mice. (D) *Cyp7a1* expression was lower because of both diet and *Mthfr* genotype. (E) Expression of the transcriptional regulator *Ppara* was lower in FASD mice. (F) *Scd1* expression was lower in FASD-fed mice. (G) *Fads2* expression was lower in FASD-fed mice and tended to be lower because of *Mthfr* genotype (*P* = 0.071). (H) *Pnpla2* (adipose triglyceride lipase) expression was lower in FASD-fed mice. White bars: *Mthfr*^+/+^; gray bars: *Mthfr*^+/−^. *n* = 4–6 per group, mean ± SEM, analyzed by 2-factor ANOVA. CD, control diet; D, diet; FASD, folic acid–supplemented diet; G, genotype; SREBP-1, sterol regulatory element-binding protein 1.

The expression of several important genes/proteins in cholesterol and lipid metabolism was evaluated (**Figure 6**). Many of these genes have been shown to be involved in NAFLD (56–59). Sterol regulatory element-binding protein (SREBP) 1 and 2 are important transcriptional regulators of genes in lipogenesis and cholesterol biosynthesis, respectively, although there is overlap between the pathways. FXR (*Nr1h4*) is a master regulator that influences the expression of genes involved in bile acid (cholesterol), lipid, and glucose metabolism (60). SREBP-1 activity is downregulated by FXR. Peroxisome proliferator-activated receptor α (PPARα) is a transcription factor in lipid metabolism that is induced by FXR (60). CYP7A1 is an FXR target that catalyzes the first and rate-limiting step in bile acid synthesis, which is a major consumer of cholesterol. SCD1 and FADS2 are lipogenic enzymes that are targets of PPARα. Adipose triglyceride lipase (ATGL; *Pnpla2*) catalyzes the first, rate-limiting step in hydrolysis of triglycerides to free fatty acids, which is required to metabolize triglycerides in lipid droplets and prevent excess lipid accumulation (57).

In FASD-fed mice, the amount of the mature SREBP-1 protein was significantly higher in the nucleus and lower in the cytoplasm (Figure 6A), indicating the activation of the SREBP-1 signaling pathway (61). The expression of *Srebp1* messenger RNA (mRNA) was not affected by diet or genotype (data not shown). Expression of *Srebp2* (Figure 6B) was higher in FASD, particularly in *Mthfr*^+/−^ mice, suggesting that this pathway is also activated.

Expression of *Nr1h4* (FXR, Figure 6C) was reduced in FASD-fed mice. An important target of FXR, *Cyp7a1*, was also reduced in expression in a significant manner for both diet and MTHFR deficiency (Figure 6D). Diminished expression of *Nr1h4* in FASD is consistent with the reduced expression of its target *Ppara* (Figure 6E) and the *Ppara* targets *Scd1* (Figure 6F) and *Fads2* (Figure 6G) in FASD-fed mice. Expression of *Pnpla2* (ATGL, Figure 6H) was also lower due to FASD. For 2 of the genes in lipid and cholesterol metabolism that were altered in expression due to diet, there was also an effect of genotype (*Cyp7a1* and *Srebp2*). These findings suggest that genotype and diet may have a cumulative effect on lipid gene expression that contributes to liver injury in FASD^+/−^ mice.

### Methylation of 2 CpG sites in the Cyp7a1 gene was higher in *Mthfr*-deficient mice

Because CYP7A1 is a critical protein in cholesterol export and its expression was dramatically reduced by diet and genotype, we assessed a potential mechanism for the altered expression. The methylation of 2 CpGs in the *Cyp7a1* gene was examined by bisulfite pyrosequencing (*n* = 6/group). Methylation was higher in *Mthfr*^+/−^ compared with *Mthfr*^+/+^ mice by a small but significant amount; there were no differences due to diet. CpG1 methylation rose from ∼70% to ∼73% (ANOVA, genotype, *P* = 0.017); CpG2 rose from ∼50% to ∼53% (ANOVA, genotype, *P* = 0.009). Although the observed changes were small, they may have contributed to the observed reduction in *Cyp7a1* expression.

### High folic acid consumption results in a proapoptotic environment in liver

The cytoplasmic clearing observed in FASD *Mthfr*^+/−^ livers may be the result of cells beginning the process of apoptosis. Therefore, measurements of *Bcl-XL* (antiapoptotic) and *Bak* (proapoptotic) mRNA and caspase 3/7 activity were performed (**Table 3**). Expression of *Bcl-XL* was significantly lower in FASD-fed mice (ANOVA, diet, *P* < 0.05), whereas *Bak* expression was higher but not significantly. The *Bcl-XL/Bak* ratio, a marker of apoptotic potential (47, 62), declined significantly (Table 3), indicating a relatively proapoptotic environment compared with CD-fed mice. Caspase 3/7 activity also tended to be higher in FASD-fed mice (ANOVA, *P* = 0.064, Table 3). Since caspase-3 can cleave SREBPs to the mature active protein (63), our finding of higher nuclear SREBP-1 is consistent with higher caspase activity.

**TABLE 3:** FASD creates a proapoptotic environment in the liver^1^

	CD		FASD		2-Factor ANOVA *P* value		
	*Mthfr*^+/+^	*Mthfr*^+/−^	*Mthfr*^+/+^	*Mthfr*^+/−^	Genotype	Diet	Interaction
*Bcl-XL/Bak*^2^	1.17 ± 0.15^3^	1.23 ± 0.12	0.91 ± 0.06	0.90 ± 0.12	0.815	0.023	0.760
Caspase 3/7^4^	41.2 ± 5.5	34.3 ± 3.9	47.6 ± 3.0	47.3 ± 6.4	0.473	0.064	0.506

1 *n* = 5–6 per group. CD, control diet; FASD, folic acid–supplemented diet.

2 Relative expression by quantitative reverse transcriptase–polymerase chain reaction.

3 Mean ± SE (analyzed by 2-factor ANOVA; all such values).

4 Activity ×1000 (relative light units).

## DISCUSSION

Concerns have been raised that excess folic acid intake may have unknown consequences (4, 5). We examined the effects of a high folic acid diet on livers of *Mthfr*-deficient mice as a model for the high folate intake in many populations due to the combination of fortification and vitamin supplementation. We have previously reported that plasma folate of mice fed this 10× RDA diet is ∼2.5-fold higher than that of mice fed the RDA (14). Plasma folate was also ∼2.5-fold higher in women given 4 mg folic acid/d (10× RDA) compared with 400 μg/d (RDA) (64). A similar increase in plasma folate was observed in the US population post-1998 due to fortified foods and vitamin supplements (65). Although plasma folate increased in FASD-fed mice, plasma homocysteine did not decrease (14), as observed in human studies (64), suggesting that not all of the ingested folic acid is metabolized.

In this study, diet and *Mthfr* genotype had significant effects on hematologic parameters. The lower RBC count, hematocrit, and hemoglobin and the higher mean corpuscular volume, particularly in FASD *Mthfr*^+/−^ mice, are consistent with reduced hematopoiesis, suggesting that FASD may impair nucleotide biosynthesis in addition to methylation reactions.

We observed cumulative effects of *Mthfr* genotype and FASD resulting in pseudo-MTHFR deficiency and reduced methylation capacity. MTHFR protein levels were lowered by the *Mthfr*^+/−^ genotype, as expected, and by FASD. The proportion of phosphorylated MTHFR, which is less active (36), was higher in FASD-fed mice. Although we confirmed that MTHFR activity is inhibited by folic acid in vitro, suggesting that UFA might inhibit MTHFR in tissues, UFA in the liver was low and unlikely to have as great an effect as the expression and phosphorylation changes. Reduced methylTHF, SAM, and SAM/SAH ratio are consequences of lower MTHFR expression and follow the same diet/genotype pattern (Figure 3). Altered methylation cycle gene expression (Figure 4) adds to the lower methylation capacity of FASD mice.

*Mthfr*^+/−^ mice are particularly dependent on betaine-dependent homocysteine remethylation (28), consistent with the reduced betaine concentration in this study. However, the increased flux through betaine was insufficient to maintain SAM concentrations. Reduced *Chdh* expression suggests that capacity for betaine synthesis may not have met betaine demand. Altered *Chdh* expression may be a mechanism to spare choline for PtdCho synthesis via the CDP-choline pathway because PEMT expression is reduced. This is consistent with observations in humans as the rate of homocysteine remethylation may be reduced in patients with nonalcoholic steatohepatitis (NASH) (66).

A major consumer of liver SAM is methylation of PE to PtdCho by PEMT (51). PtdCho synthesis via the CDP-choline pathway may be upregulated to compensate for reduced SAM concentration and *Pemt* expression, consistent with the reduction in PCho. Lower concentrations of PCho, PtdCho, SM, and LysoPtdCho in *Mthfr*^+/−^ mice suggest that reduced methylation capacity due to MTHFR deficiency leads to greater use of betaine as a one-carbon donor and affects the choline-dependent synthesis of membrane phospholipids.

PtdCho is an important component of the plasma membrane and VLDL. Reduced PtdCho synthesis can result in liver injury through at least 2 mechanisms. Altered PtdCho synthesis is associated with lipid accumulation caused by impaired secretion of lipid via VLDL particles (51, 67). VLDL particles and lipid secretion are abnormal in *Mat1a*^−/−^ mice, which have reduced SAM and develop NAFLD/NASH (68). Disrupted PtdCho synthesis may also alter membrane fluidity and integrity, which can damage hepatocytes. Reduced PtdCho/PE ratios cause membrane leakage, leading to steatohepatitis in *Pemt*^−/−^ mice, and are associated with NASH in humans (69). Altered membrane integrity leads to hydropic degeneration and hepatocyte ballooning (50, 69), forms of degeneration observed in FASD *Mthfr*^+/−^ livers. Reduced PtdCho and *Pemt* expression in FASD^+/−^ mice may have resulted in altered membrane integrity, contributing to liver injury.

Other major mechanisms by which liver damage arises in NAFLD/NASH are accumulation of lipids due to increased synthesis, reduced mobilization of fatty acids from lipid droplets, and cholesterol accumulation (56, 57). We evaluated a panel of genes in these pathways, including the major regulators SREBP-1, SREBP-2, and FXR (*Nr1h4*). Dysregulation of SREBP-1 and SREBP-2 is commonly observed in NAFLD/NASH. PtdCho is a regulator of SREBP-1 (61); the reduction in PtdCho in FASD *Mthfr*^+/−^ mice would be expected to activate SREBP-1, which would upregulate lipogenic pathways. We observed greater SREBP-1 in the nucleus and greater SREBP-2 mRNA associated with FASD. FXR is a bile acid receptor that regulates genes involved in the production of bile acid from cholesterol. FXR activity, which is inversely related to severity of steatosis in patients with NAFLD (60), downregulates *Srebp1* and, indirectly, *Cyp7a1* (56, 60). Reduced *Nr1h4* in FASD-fed mice may have led to greater nuclear SREBP-1. FXR has also been shown to upregulate *Ppara* (60, 70). Reduced expression of *Ppara* and its targets *Scd1* and *Fads2* is consistent with lower *Nr1h4* expression. Expression of *Pnpla2* (ATGL) was also lower in FASD-fed mice. ATGL catalyzes the first step of triglyceride hydrolysis to generate free fatty acids; triglyceride hydrolysis products upregulate PPARα signaling (71). Disruption of *Pnpla2* prevents extraction of free fatty acids from lipid droplets, leading to severe steatosis and hepatic inflammation in mice (57, 72).

CYP7A1 catalyzes the rate-limiting step in bile synthesis, a major consumer of cholesterol; downregulation of CYP7A1 lowers cholesterol catabolism, raising cholesterol levels (60). Both FASD and the *Mthfr*^+/−^ genotype significantly lowered *Cyp7a1* expression. Greater methylation of *Cyp7a1* CpGs in *Mthfr*^+/−^ mice may have contributed to this effect. Reduced CYP7A1 may result in a buildup of intracellular cholesterol, leading to toxic effects such as altered membrane integrity; reduced CYP7A1 is associated with greater severity of NASH (56).

Altered lipid gene expression, activation of SREBP-1, and disturbed PtdCho metabolism suggest that lipid accumulation may be involved in the hepatocyte degeneration in FASD *Mthfr*^+/−^ mice. The heavier livers are consistent with lipid accumulation [as in other models (for review, see da Silva et al. (51)] and enlargement of degenerating cells. Greater liver weights correlated with the presence of widespread degenerating hepatocytes (Pearson correlation *r* = 0.502, 2-tailed *P* = 0.006). FASD also induced a proapoptotic environment, which may predispose FASD *Mthfr*^+/−^ hepatocytes to enter apoptosis in response to injury.

FASD may cause epigenetic changes due to reduced SAM and aberrant DNA methylation that could affect health [reviewed in Smith (4)]. MTHFR deficiency in humans and mice is associated with altered DNA methylation (32, 73). Methylation changes could influence expression of a variety of genes. We observed greater methylation of 2 CpGs in *Cyp7a1* that could contribute to its reduced expression in *Mthfr*^+/−^ mice. Similarly, Devlin et al. (58) reported greater methylation in *Fads2* associated with reduced *Fads2* mRNA in mice fed a low-folate, high-methionine diet.

In conclusion, high folic acid consumption in mice results in a state of MTHFR deficiency, with reduced methylTHF and methylation capacity. Findings in murine studies may not be directly extrapolated to the human situation, but they suggest that additional studies are warranted, given the increased folate intake in many populations. Many of the dramatic effects on choline metabolites and gene expression were due to the combination of MTHFR deficiency and FASD. These findings suggest a 2-hit mechanism whereby MTHFR-deficient hepatocytes are less able to mitigate the effect of phospholipid and lipid disturbances, leading to hepatocyte injury. MTHFR deficiency in hepatocytes may set the stage for other insults (e.g., high-fat diets) that could also lead to NAFLD. Further study is necessary because the small sample size may be insufficient to detect important genotype-diet interactions, and the lack of correction for multiple testing in this preliminary report raises the possibility of type I error. Nonetheless, these results may have implications for the human population, as genotype-diet interactions are known to modulate disease risk in *MTHFR* 677 C>T individuals (31). It may be important to consider variation in folate-related genes when making recommendations for folic acid intake at both the high and low ends of the dietary folate spectrum.

## Supplementary Material

Supplemental data

## Footnotes

6 Abbreviations used: *Actb*, β-actin; ATGL, adipose triglyceride lipase; *Bak*, BCL2-antagonist/killer 1; *Bcl-XL*, Bcl2-like 1; *Bhmt*, betaine-homocysteine methyltransferase; *B2m*, β-2-microglobulin; CD, control diet; *Chdh*, choline dehydrogenase; *Cyp7a1*, cytochrome P450, family 7, subfamily a, polypeptide 1; *Fads2*, fatty acid desaturase 2; FASD, folic acid–supplemented diet; FXR, farnesoid X receptor; LysoPtdCho, lysophosphatidylcholine;* Mat1a*, methionine adenosyltransferase I, α; mRNA, messenger RNA; MTHFR, methylenetetrahydrofolate reductase; *Mtr*, methionine synthase; NAFLD, nonalcoholic fatty liver disease; NASH, nonalcoholic steatohepatitis; *Nr1h4*, nuclear receptor subfamily 1, group H, member 4; PCho, phosphocholine; PE, phosphatidylethanolamine; PEMT, phosphatidylethanolamine N-methyltransferase; *Pnpla2*, patatin-like phospholipase domain containing 2; PPARα, peroxisome proliferator-activated receptor α; PtdCho, phosphatidylcholine; RBC, red blood cell; RDA, Recommended Dietary Allowance; RT-PCR, reverse transcriptase–polymerase chain reaction; SAH, S-adenosylhomocysteine; SAM, S-adenosylmethionine; *Scd1*, stearoyl–coenzyme A desaturase 1; *Sdha*, succinate dehydrogenase; SM, sphingomyelin; SREBP, sterol regulatory element-binding protein; THF, tetrahydrofolate; UFA, unmetabolized folic acid; *Ywhaz*, tyrosine 3-monooxygenase/tryptophan 5-monooxygenase activation protein, zeta polypeptide.
