# Supplementary material for: High folic acid consumption leads to pseudo-MTHFR deficiency, altered lipid metabolism, and liver injury in mice1
Source: Am J Clin Nutr. 2015 Jan 7;101(3):646–58. doi: 10.3945/ajcn.114.086603 (PMC4340065; doi:10.3945/ajcn.114.086603)
Supplement: Supplemental data [file supp_101_3_646__index.html]

High folic acid consumption leads to pseudo-MTHFR deficiency, altered lipid metabolism, and liver injury in mice — Supplemental data 

# High folic acid consumption leads to pseudo-MTHFR deficiency, altered lipid metabolism, and liver injury in mice

## Supplemental data

**Files in this Data Supplement:**

- Supplemental data - Figure 1
- Supplemental data - Table 1
- Supplemental data - Table 2
