# Supplementary material for: High folic acid consumption leads to pseudo-MTHFR deficiency, altered lipid metabolism, and liver injury in mice1
Source: Am J Clin Nutr. 2015 Jan 7;101(3):646–58. doi: 10.3945/ajcn.114.086603 (PMC4340065; doi:10.3945/ajcn.114.086603)
Supplement: Supplemental data [file 114.086603_ajcn086603SupplementaryData1.pdf]

Online Supplemental Material

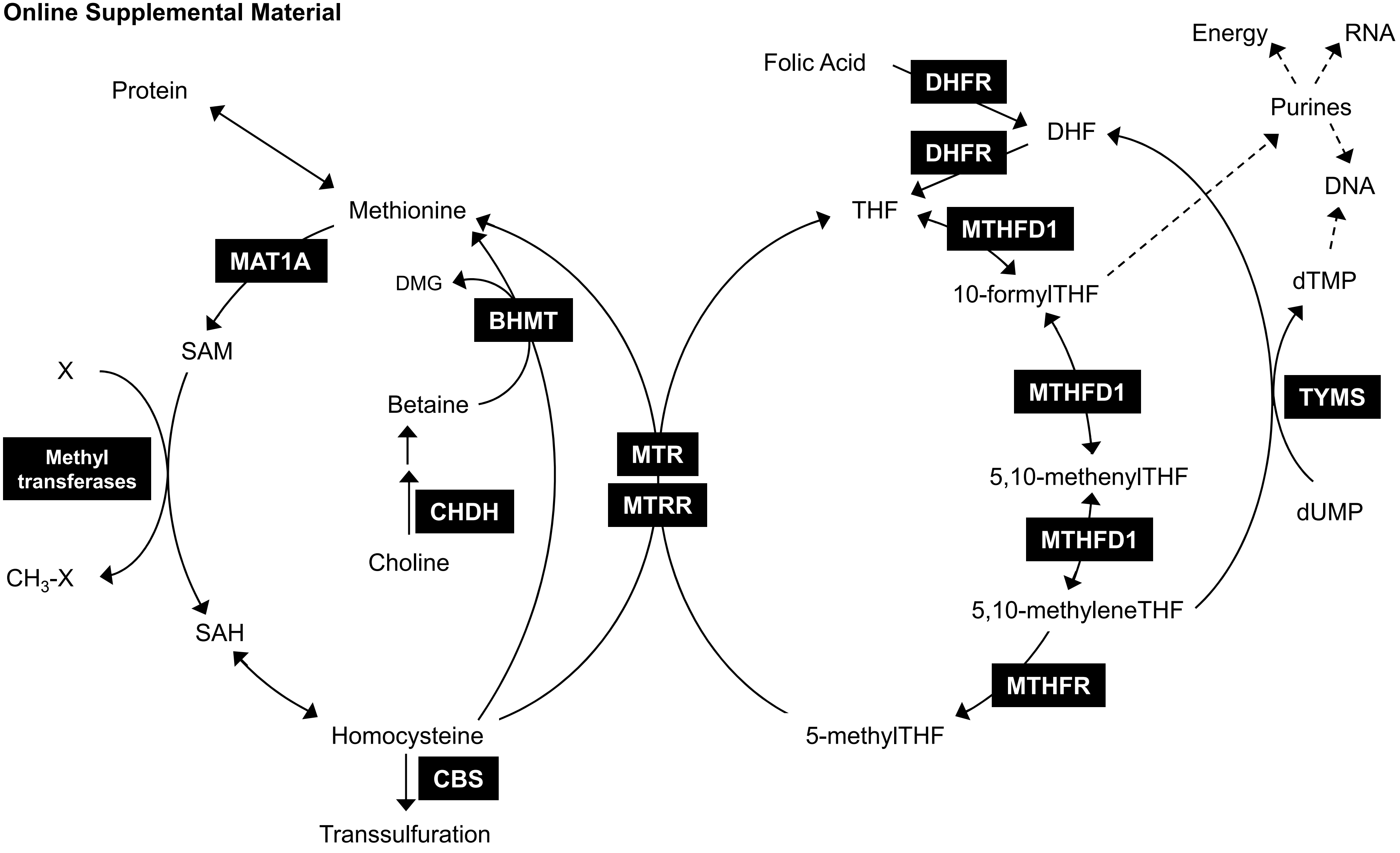

Supplemental Figure 1: One-carbon folate metabolism. MTHFR catalyzes the irreversible reduction of methyleneTHF to methylTHF, supplying carbon to support methylation reactions via SAM.

Abbreviations: BHMT: betaine-homocysteine methyltransferase; CBS: cystathionine beta-synthase; CHDH: choline dehydrogenase; DHF: dihydrofolate**;** DHFR: DHF reductase; DMG: dimethylglycine; MTHFD1: methyleneTHF dehydrogenase-methenylTHF cyclohydrolase-formylTHF synthetase; MAT1A: methionine adenosyltransferase 1; MTHFR: methylTHF reductase; MTR: methionine synthase; MTRR: MTR reductase; SAH: S-adenosylhomocysteine; SAM: S-adenosylmethionine; THF: tetrahydrofolate; TYMS: thymidylate synthase
