# Supplementary material for: High folic acid consumption leads to pseudo-MTHFR deficiency, altered lipid metabolism, and liver injury in mice1
Source: Am J Clin Nutr. 2015 Jan 7;101(3):646–58. doi: 10.3945/ajcn.114.086603 (PMC4340065; doi:10.3945/ajcn.114.086603)
Supplement: Supplemental data [file 114.086603_ajcn086603SupplementaryData2.pdf]

Online Supplemental Material

Supplemental Table 1: Primers for quantitative real-time PCR

Gene Symbol	*Gene Name*	*Sequence*	*Primer Sequence*	*Amplicon Size (bp)*	*Tann (°C)*	*[Primer] (μmol/L)*	*50 mmol/L $MgCl_2$/rxn*
Bak	BCL2-antagonist/killer 1	NM_007523.2	TATTAACCGGCGCTACGACAC<br>CTTAAATAGGCTGGAGGCGATCTT	109	60	0.4	
Bcl-XL	BCL2-like 1<br>(all transcripts)	NM_001289717.1	GGTAGTGAATGAACTCTTTCGGGAT<br>TCCGACTCACCAATACCTGCAT	131	60	0.4	
Bhmt	betaine-homocysteine methyltransferase	NM_016668.3	TCCACCCATCTAGGAGACCAATCT<br>ACACGCCCACCAGCCTTTGAATTA	91	60	0.4	
Chdh	choline dehydrogenase<br>(all transcripts)	NM_175343.3	TGGCTGAAACTGAGAAGTGCCAAC<br>TTTCTCGTGACAGCCTCACACACT	115	60	0.2	
Cyp7a1	cytochrome P450, family 7, subfamily a, polypeptide 1	NM_007824.2	ACTTCTGCGAAGGCATTTGG<br>GAGCATCTCCCTGGAGGGTT	103	62	0.4	
Fads2	fatty acid desaturase 2	NM_019699.1	TCATCGACCGCAAGGTCTAC<br>AGGCATCCGTAGCATCTTCTC	102	64	0.2	1.5 µL
Mat1a	methionine adenosyltransferase I, alpha	NM_133653.3	TGGTGAGAGACACCATCAAGCACA<br>TTGCTCCAGAGCCACTAGCACATT	92	60	0.4	1.5 µL
Mtr	methionine synthase	NM_001081128.2	ACTCATGGCACAGGAGGGAAGAAA<br>TGCCCTTCACAAGAGCATACTCCA	94	60	0.4	
Nr1h4	nuclear receptor subfamily 1, group H, member 4<br>(farnesoid X receptor, all transcripts)	NM_001163504.1	CTGAGACTGGGTACCAGGGA<br>CCATTCGCGGCTTCTTTGTC	66	62	0.6	
Pemt	phosphatidylethanolamine N-methyltransferase	NM_008819.2	GAATGTGGTAGCGAGATGGGA<br>GGGAGCGGAGGATGTTCAAA	119	62	0.4	1.5 µL
Pnpla2	patatin-like phospholipase domain containing 2<br>(both transcripts)	NM_001163689.1	GCCACTCACATCTACGGAGC<br>TTGGCACCTGCTTCACCCAG	86	64	0.4	1.5 µL
Ppara	peroxisome proliferator activated receptor alpha<br>(both transcripts)	NM_001113418.1	CTGGGCAAGAGAATCCACGA<br>GACAAAAGGCGGGTTGTTGC	105	62	0.4	
Scd1	stearoyl-Coenzyme A desaturase 1	NM_009127.4	CACTGGGAAAGTGAGGCGAG<br>GAACTGGAGATCTCTTGGAGCA	70	60	0.4	
Srebp1	sterol regulatory element binding transcription factor 1	NM_011480.3	GGAACAGACACTGGCCGAGAT<br>ATGAGCTGGAGCATGTCTTCGAT	75	62	0.4	
Srebp2	sterol regulatory element binding transcription factor 2	NM_033218.1	AGAGGCGGACAACACACAAT<br>GCCAGACTTGTGCATCTTGG	116	62	0.4	
Actb	beta-actin	NM_007393.3	CTGACGGCCAGGTCATCACTA<br>TAGTTTCATGGATGCCACAGGAT	105	60	0.4	
B2m	beta-2 microglobulin	NM_009735.3	ATGCTATCCAGAAAACCCCTCAA<br>GCGGGTGGAACTGTGTTACG	100	60	0.4	
Gapdh	glyceraldehyde-3-phosphate dehydrogenase<br>(all transcripts)	NM_008084.3	CAGGAGCGAGACCCCACTAACAT<br>AAGACACCAGTAGACTCCACGAC	74	62	0.6	
Sdha	succinate dehydrogenase	NM_023281.1	GCTGTGGCCCTGAGAAAGATC<br>ATCATGGCCGTCTCTGAAATTC	100	60	0.4	
Ywhaz	tyrosine 3-monooxygenase/tryptophan<br>5-monooxygenase activation protein, zeta polypeptide	NM_011740.2	TGCTGGTGATGACAAGAAAGGA<br>TGAGGGCCAGACCCAGTCT	119	60	0.4	
