# Supplementary material for: High folic acid consumption leads to pseudo-MTHFR deficiency, altered lipid metabolism, and liver injury in mice1
Source: Am J Clin Nutr. 2015 Jan 7;101(3):646–58. doi: 10.3945/ajcn.114.086603 (PMC4340065; doi:10.3945/ajcn.114.086603)
Supplement: Supplemental data [file 114.086603_ajcn086603SupplementaryData3.pdf]

Online Supplemental Material

Supplemental Table 2: Measurement of hepatic folate and choline metabolites in *Mthfr*$^{+/+}$ and *Mthfr*$^{+/-}$ mice fed CD and FASD[1]

	CD		FASD		2-way ANOVA p-value		
	Mthfr$^{+/+}$	*Mthfr*$^{+/-}$	*Mthfr*$^{+/+}$	*Mthfr*$^{+/-}$	Diet	Genotype	Interaction
**Folates (% total folates)**							
DHF	1.13±0.24	1.27±0.03	1.49±0.18	1.13±0.17	0.523	0.531	0.159
THF	60.0±3.1 [a]	71.0±3.0 [a]	67.5±3.5 [a]	56.6±9.2 [a]	0.521	0.997	**0.058**
methenylTHF	0.81±0.18	1.25±0.21	1.49±0.32	1.48±0.44	0.159	0.495	0.479
methyleneTHF	2.77±0.21	2.90±0.18	4.11±1.21	2.72±0.63	0.392	0.356	0.268
methylTHF	27.7±1.1	19.3±3.6	17.0±1.4	16.8±2.2	***0.018***	0.098	0.115
formylTHF	3.31±0.55	4.25±0.29	4.95±0.67	5.75±2.33	0.228	0.497	0.955
folic acid	0.042±0.007	0.036±0.005	0.060±0.009	0.064±0.020	**0.074**	0.981	0.690
**Choline Metabolites (nmol/g)**							
SAM	60.5±7.0	42.7±7.3	40.1±6.9	35.3±4.2	***0.048***	0.101	0.333
SAH	46.9±4.5 [a]	66.2±1.5 [a]	66.8±8.1 [a]	48.6±4.8 [a]	0.835	0.917	***0.005***
SAM/SAH	1.36±0.22 [a]	0.70±0.14 [a,Δ]	0.68±0.18 [a,Δ]	0.78±0.15 [a]	0.103	0.131	***0.046***
methionine	51.4±3.0	44.0±4.3	57.2±7.6	47.4±3.8	0.367	0.106	0.818
choline	297±48	277±39	267±27	203±51	0.236	0.331	0.611
betaine	77.4±10.0 [a,b]	116±19 [a]	96.3±7.1 [a]	34.4±7.2 [b]	***0.022***	0.361	***0.001***
GPC	566±93	494±58	499±81	599±52	0.800	0.848	0.256
PCho	375±9	300±58	464±64	230±54	0.856	***0.008***	0.139
PtdCho	29735±556 [a,b]	28110±605 [b,c]	31139±461 [a]	26143±253 [c]	0.591	***0.00001***	***0.005***
SM	638±19 [a,b]	606±22 [a]	732±29 [b]	591±32 [a]	0.153	***0.004***	**0.053**
LysoPtdCho	226±7	214±18	279±32	207±18	0.286	**0.057**	0.169

[1] n = 4-5 per group, mean ± standard error

Significant ANOVA p-values are bold and italicized, borderline values are bold.

Means in a row without a common letter are significantly different by Tukey post-hoc, $p < 0.05$

[Δ] = borderline significant difference from CD$^{+/+}$, Tukey post-hoc p-value 0.051 – 0.075

Abbreviations: DHF – dihydrofolate, THF – tetrahydrofolate, SAM – S-adenosylmethionine, SAH – S-adenosylhomocysteine, GPC – glycerophosphocholine, PCho – phosphcholine, PtdCho – phosphatidylcholine, SM – sphingomyelin.
